# Transcriptional profiling identifies critical steps of cell cycle reprogramming necessary for *Plasmodiophora brassicae*‐driven gall formation in Arabidopsis

**DOI:** 10.1111/tpj.14156

**Published:** 2019-01-05

**Authors:** Marcin Olszak, William Truman, Karolina Stefanowicz, Elwira Sliwinska, Masaki Ito, Piotr Walerowski, Stephen Rolfe, Robert Malinowski

**Affiliations:** ^1^ Department of Integrative Plant Biology Institute of Plant Genetics of the Polish Academy of Sciences ul. Strzeszyńska 34 60‐479 Poznań Poland; ^2^ Laboratory of Molecular Biology and Cytometry Department of Plant Genetics, Physiology and Biotechnology UTP University of Science and Technology Kaliskiego Ave. 7 85‐789 Bydgoszcz Poland; ^3^ Graduate School of Bioagricultural Sciences Nagoya University Chikusa Nagoya 464‐8601 Japan; ^4^ Department of Animal and Plant Sciences University of Sheffield Sheffield S10 2TN UK

**Keywords:** *Plasmodiophora brassicae*, clubroot, *Arabidopsis thaliana*, cell cycle reprogramming, cell division

## Abstract

*Plasmodiophora brassicae* is a soil‐borne biotroph whose life cycle involves reprogramming host developmental processes leading to the formation of galls on its underground parts. Formation of such structures involves modification of the host cell cycle leading initially to hyperplasia, increasing the number of cells to be invaded, followed by overgrowth of cells colonised by the pathogen. Here we show that *P. brassicae* infection stimulates formation of the E2Fa/RBR1 complex and upregulation of *MYB3R1*,*MYB3R4* and A‐ and B‐type cyclin expression. These factors were previously described as important regulators of the G2−M cell cycle checkpoint. As a consequence of this manipulation, a large population of host hypocotyl cells are delayed in cell cycle exit and maintained in the proliferative state. We also report that, during further maturation of galls, enlargement of host cells invaded by the pathogen involves endoreduplication leading to increased ploidy levels. This study characterises two aspects of the cell cycle reprogramming efforts of *P. brassicae*: systemic, related to the disturbance of host hypocotyl developmental programs by preventing cell cycle exit; and local, related to the stimulation of cell enlargement via increased endocycle activity.

## Significance Statement

We describe the changes in host cell cycle gene expression in response to *P. brassicae* infection, and characterise the impact of key cell cycle regulators (MYB3R4, E2Fa, CCS52A1), establishing that cell proliferation during gall development stems from repression of cell cycle exit, whereas hypertrophy in later disease stages depends on stimulation of endoreduplication. This provides a molecular framework for understanding the progress of clubroot disease and gall formation.

## Introduction

*Plasmodiophora brassicae* is a pathogenic protist currently recognised as a major world‐wide threat to oilseed rape production as well as a notorious problem in the cultivation of other brassicas. Its life cycle can be divided into three main stages (Kageyama and Asano, 2009): (i) primary infection of root‐hair cells and subsequent development of secondary zoospores, which are released into the soil; (ii) penetration of host cortical tissues by these secondary zoospores, leading to secondary infection and gall formation; and (iii) maturation of resting spores that will be released into the soil upon the death of the plant and disintegration of root material.

Galls are the main sites of *P. brassicae* accumulation and the principal source of resting spores − capable of surviving and retaining infectivity in the soil for many years. Post‐infection developmental reprogramming of the host giving rise to gall formation may have far‐reaching consequences on the amount of resting spores ultimately released to the soil. Therefore, understanding the molecular bases of reprogramming underpinning the development of these structures is a critical step for future strategies of clubroot disease management. The purpose of this study was to refine our understanding of the manipulation of host cell cycle machinery induced by *P. brassicae* in its efforts to subvert host development programs.

Plant galls are unusual structures formed as a consequence of cell cycle and cell growth disruption or reprogramming. Typically they occur as a consequence of fungal, bacterial, nematodal, viral or insect attacks leading to an increase in cell proliferation and local change in morphogenesis. The precise mechanism by which cell division and further morphogenesis is modified is very complex and varies with different species interactions and plant tissue types. The induction of cell proliferation by *P. brassicae* in Arabidopsis roots at early stages of infection was previously observed with the use of B‐type cyclin (*CYCB1::GUS‐CYCB1*) reporter line by Devos *et al*. (2006). Our previous work further established a qualitative and quantitative assessment of changes in meristematic activity during clubroot‐driven gall formation (Malinowski *et al*., 2012). This work challenged the existing view that galls are built as a result of *de novo* meristematic activity, instead we were able to show that an increase in cambium proliferation is crucial for this process. Further understanding of this developmental reprogramming needs, however, detailed study of host cell cycle progression.

The cell cycle in plants is regulated in a complex manner and factors directly involved in this process have been designated the core cell cycle genes/proteins. Core cell cycle regulators influence the timing and duration of particular cell cycle phases and are involved in both monitoring and determination of cell fate. Major control, influencing cell fate during mitosis, occurs between the G1−S and G2−M phases (Gutierrez, 2009). The G1−S checkpoint influences nucleic acid replication and links the cell cycle to external signals, whereas the G2−M checkpoint influences duration and maintenance of the proliferative state and subsequent mitotic spindle formation. Frequently both checkpoints additively influence cell fate, controlling processes like endoreduplication, cell death and differentiation (Scofield *et al*., 2014). Recent advances in our understanding of how progress from phase to phase is controlled have implicated the so‐called DREAM complex as a key regulatory component. Analogous to the DREAM complex characterised as regulating cell cycle progression in animals, in Arabidopsis a complex comprising various combinations of DPa, MYB3R and E2F transcription factors (TFs) and RBR1 was described (Magyar *et al*., 2016). An extensive amount of information regarding the activity of core cell cycle genes and networks comes from studies on synchronised cells of the BY2 tobacco line (Menges and Murray, 2002; Menges *et al*., 2005). Such an approach cannot be directly applied to plant organs as they are typically composed of a population of non‐synchronised cells frequently with a mixture of different ploidy levels. In order to facilitate further interpretation we have opted to analyse two distinct cellular stages of *P. brassicae*‐driven gall formation, choosing times within these stages in which the maximum synchronisation of developmental processes can be observed. The gall formation process begins with a transient increase in cell proliferation (7–18 days after infection) followed by an abrupt cessation of cell divisions and an increase in cell size leading to the formation of hypertrophied cells (21–32 days after infection). Samples collected during the first phase are significantly enriched for cells in a proliferative state, while the latter stage is typified by cells that have exited the cell cycle. In our experiments we aimed to discover the patterns of core cell cycle gene expression that define the proliferative stage of gall formation. We found that *P. brassicae* infection manipulates a major mechanism responsible for exit from the cell cycle and commencement of endoreduplication. Proliferating galls had increased levels of B‐type cyclins known to be involved in the regulation of G2 phase duration, entry into mitosis and transition to anaphase. Functional characterisation of the roles of DREAM complex components MYB3R4 and E2Fa confirmed the importance of G2−M‐specific reprogramming for gall formation. At later stages of gall development *P. brassicae* infection locally induces the endoreduplication process, manifested by the formation of hypertrophied cells. Our results are discussed in light of the hypothesis that critical events related to the manipulation of host cell cycle for gall formation revolve around the G2−M checkpoint which, depending on the stage of disease development, acts as a switch between promoting host cell proliferation versus driving polyploidy to facilitate hypertrophy in pathogen‐colonised cells.

## Results

### Core cell cycle gene expression reveals clear distinctions between the proliferative and expansive stages of gall development

**Figure 1:**
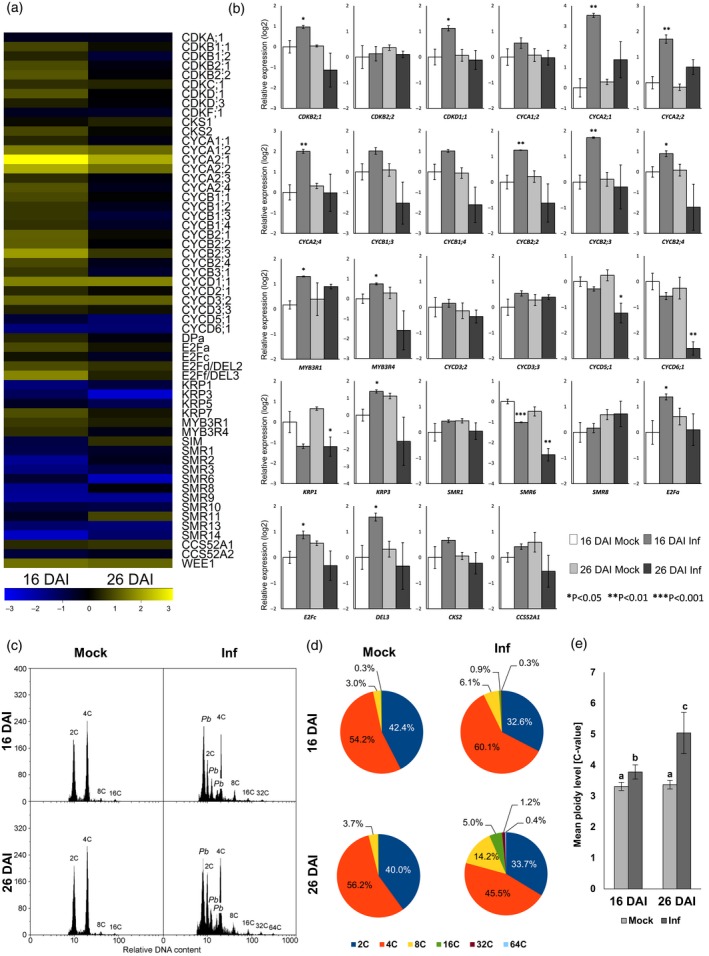
Changes in core cell cycle gene expression triggered by *P. brassicae* infection correlate with cellular DNA content and reflect cellular patterns during gall formation. (a) Heat maps showing changes in expression of core cell cycle genes with significant differences between levels in mock and *P. brassicae*‐infected hypocotyls at either 16 or 26 days after inoculation (DAI) defining, respectively the proliferative and expansive stages of gall development. Yellow indicates upregulation in the infected tissue relative to controls while blue represents downregulation, the colour bar corresponds to log (base 2) ratios. Results were obtained from RNA‐seq profiling of three independent biological replicates. Significance of differential expression was based on FDR < 0.05, numerical data are presented in Table S1. (b) qRT‐PCR validation of the *P. brassicae*‐induced changes in expression of selected core cell cycle genes identified from RNA‐seq profiling. Means of the relative expression, normalised to the 18S rRNA housekeeping gene, are plotted with the standard error. Three independent biological replicates were used, each with 30 plants per treatment. Statistically significant differences between uninfected and infected plants determined by Student's *t*‐test are indicated as follows: *P < 0.05, **P < 0.01, ***P < 0.001. (c) Histogram of DNA content determined by flow cytometry in nuclei isolated from infected and uninfected hypocotyls of Col‐0 plants 16 and 26 DAI. The peaks corresponding to various host ploidy levels are labelled as are *P. brassicae* peaks. (d) Percentage of nuclei with different DNA contents isolated from the mock‐inoculated or infected hypocotyls of Col‐0 plants 16 and 26 DAI. These values are the average of 12 replicates for each condition. (e) Mean ploidy level of the nuclei isolated from the hypocotyls of mock and infected Col‐0 plants 16 and 26 DAI. Error bars indicate the standard error, different letters denote statistically significant differences at *P* < 0.05 (Duncan's test).

In order to evaluate the effect of *P. brassicae* on cell cycle progression, samples collected from two distinct time‐points of disease progression were taken. The first time‐point, 16 days after inoculation (DAI), exemplifies the proliferative stage (7–18 DAI) when the cells within hypocotyls of infected plants are driven to proliferate. The second time‐point, 26 DAI, represents the expansive stage (21–32 DAI) when hypocotyl cells are expanding and severe pathogen‐driven hypertrophy occurs. These two time‐points were selected following detailed time‐course studies of *CYCB1* expression in GUS reporter lines and microscopic observation of disease progression to best represent the proliferative and expansive phases (Malinowski *et al*., 2012). Transcript levels were profiled in RNA pools isolated from infected and non‐infected samples using RNA‐sequencing (RNA‐seq) by Illumina HiSeq (Figure 1a and Table S1). Expression patterns of core cell cycle genes differ greatly between 16 DAI and 26 DAI. Our profiling showed significant upregulation of transcripts stimulating cell proliferation at 16 DAI, this pattern is not observed at later stages (26 DAI) when cell expansion occurs (Figure 1). The largest group of core cell cycle genes affected in infected hypocotyls at 16 DAI were G2−M checkpoint regulators, in particular A‐type and B‐type cyclins. These genes are responsible for the G2−M transition and maintenance of the mitotic/proliferative state of cells (Menges *et al*., 2005). Both these groups of genes have been described as G2−M‐specific, however CYCA2 group factors are also involved in the preparation of cells to M phase entry whereas the CYCB2 group maintain cell proliferation by blocking entry to endoreduplication (Roudier *et al*., 2003). A‐type cyclins were previously characterised as important factors involved in stimulation of cell divisions in leaves (Boudolf *et al*., 2009) and proliferation of vascular cells (Donner and Scarpella, 2013). Accumulation of the B‐type cyclin transcripts upon infection was almost exclusive to 16 DAI, whereas upregulation of some A‐type cyclins was maintained at 26 DAI. Hyperplasy of hypocotyls at 16 DAI was further evidenced by increased levels of the transcription factor *E2Fa* and B‐type cyclin‐dependent kinases (Figure 1a). These genes have been characterised as factors negatively regulating endocycle onset and stimulating maintenance of the proliferative status of cells (De Veylder *et al*., 2002; Boudolf *et al*., 2009). Other genes differentially expressed in developing galls 16 DAI were two *MYB3R* TFs (*MYB3R1* and *MYB3R4*), both upregulated, that have also previously been characterised as G2−M checkpoint‐specific repressors of cell cycle exit, involved in maintenance of cell proliferation (Haga *et al*., 2011). In line with the upregulation of mitosis promoting factors, the downregulation of previously described mitosis inhibitors such as *KRP* (Nakai *et al*., 2006) and *SMR* genes (Van Leene *et al*., 2010), vital for negative regulation of cell cycle progression, were also part of the response to *P. brassicae* infection 16 DAI (Figure 1a).

The transcriptomic approach also revealed stimulation of G1−S‐specific D3‐type cyclins (*CYCD3;2* and *3;3*) upon infection 16 DAI, however these were not confirmed with qRT‐PCR (Figure 1b). Surprisingly, we found the expression of the G1−S‐specific CYCD3;1 gene was not significantly affected by infection, despite being known to be a key factor in the increase in proliferative cell status during secondary root thickening (Figure S1) (Randall *et al*., 2015). An additional effort to examine CYCD3;1 protein levels also indicated no changes in accumulation occurring upon *P. brassicae* infection (Figure S1).

During the proliferative stage of gall formation (16 DAI) we also found increased levels of transcription regulatory complex components *DEL2* and *DEL3*. Action of these genes relates to two distinct mechanisms of cell growth; *DEL2* negatively regulates anaphase promoting complex factor *CCS52A2* (Lammens *et al*., 2008) which was downregulated 16DAI; whereas *DEL3* is involved in repression of expansin genes (Ramirez‐Parra *et al*., 2004).

At 26 DAI the developmental pattern changes and cells within the gall cease proliferation (Malinowski *et al*., 2012). This was reflected in the transcriptome with a reduced magnitude in the induction of A‐type cyclins at 26 DAI compared with 16 DAI, while several B‐type cyclins as well as cyclin‐dependent kinases were actually significantly downregulated at later stages of infection (Figure 1a). RNA‐Seq analysis also indicated elevated levels of *CCS52a1* and *WEE1* transcripts at 26 DAI. These factors are known to be involved in regulating the timing of cell differentiation and are connected to DNA replication processes including endocycling (Larson‐Rabin *et al*., 2009; Cools *et al*., 2011). The upregulation of *WEE1* at 16 DAI, when proliferation dominates, may indicate that it is induced as a feedback to limit excessive proliferation, or it may be due to the heterogenous nature of infection with some early colonised cells already being driven to hypertrophy.

Sequencing results for selected core cell cycle genes showing significant up‐ or downregulation upon infection have been additionally assessed by qRT‐PCR (Figure 1b). This data confirmed the general tendency for accumulation of transcripts of G2−M‐specific mitotic status stimulating factors in *P. brassicae* infected hypocotyls at 16 DAI. In particular, for the proliferative stage of gall development (16 DAI), increased expression of G2−M‐related factors maintaining cells in a mitotic state (*CYCA2;1*,* 2;2*,* 2;4*,* CYCB2;2*,* 2;3*,* 2;4*,* CDKB2;1*,* CDKD1;1*,* E2Fa*,* MYB3R1*,* MYB3R4* and *DEL3*), as well as downregulation of the mitotic inhibitor *SMR6*, was confirmed. Comparison of responses to *P. brassicae* determined by RNA‐seq or qRT‐PCR exhibited a reasonably strong correlation (Figure S2).

In parallel with transcriptional studies, we have assessed the nuclear DNA content of cells within galls at 16 and 26 DAI. Our flow cytometric analysis showed an increased proportion of 4C nuclei at 16 DAI compared with uninfected plants (Figure 1c–e). Due to the limitations of the flow cytometry method, which does not allow us to score mitotic cells that have not yet synthesised a nuclear envelope, some 4C fraction is missing from our calculations. Nevertheless, the observed increase in the 4C fraction reflects the proliferation‐stimulating transcriptional changes observed with *P. brassicae* infection 16 DAI. At 26 DAI the 4C proportion considerably decreased. Surprisingly, at 16 DAI we observed an increased proportion of 8C and 16C nuclei, as well as some nuclei possessing 32C (having undergone additional endocycles), that did not occur in uninfected plants and the average ploidy level of the infected hypocotyls was significantly higher (Figure 1e). This increase was much more pronounced in hypocotyls at 26 DAI, when cell proliferation stopped and hypertrophied cells developed. At this stage a small fraction of nuclei underwent two additional endocycles and about 0.4% nuclei had 64C DNA.

The increased proportion of 4C nuclei together with increased expression of *E2Fa* as well as B‐type cyclins at 16 DAI suggests that enhanced cell proliferation may be an effect of disturbance in pathways controlling the duration of the mitotic state and its eventual exit necessary for cell growth or differentiation. Some individual cells however undergo endoreduplication already at this stage that can be observed as the appearance of the 32C nuclear fraction.

### E2Fa action is vital for the *P. brassicae*‐driven maintenance of a proliferative cell state

**Figure 2:**
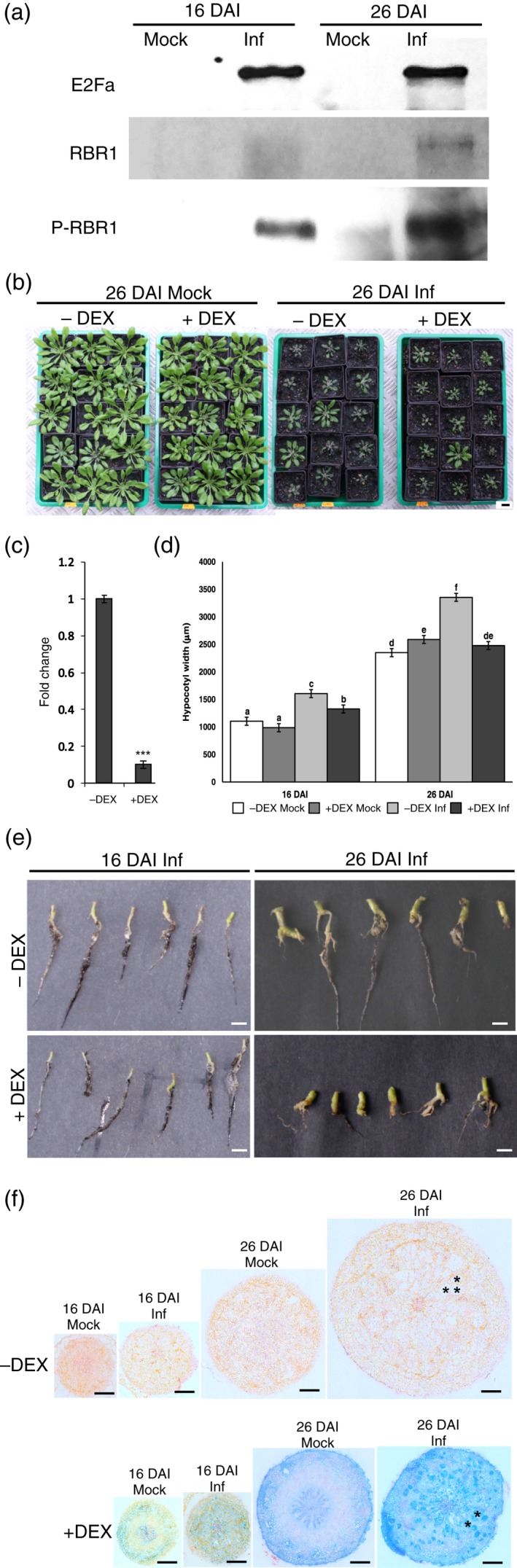
The E2Fa – RBR1 complex is involved in cell fate regulation during *P. brassicae* infection. (a) E2Fa and RBR1 levels observed in Co‐IP experiments in both the proliferative (16 DAI) and expansive (26 DAI) stage of gall formation. (b) Phenotype of the *pOpON::amiRNA‐E2Fa* plants with (Inf) and without (Mock) infection; *E2Fa* silencing was induced with dexamethasone (+DEX) from the time of *P. brassicae* inoculation 14 days post germination. (c) qRT‐PCR confirmation of the reduction in *E2Fa* transcript levels in +DEX *pOpON::amiRNA‐E2Fa* plants. (d) Size of galls formed on *P. brassicae* infected hypocotyls with DEX induced silencing of *E2Fa* in the *pOpON::amiRNA‐E2Fa* line. Means and variance of hypocotyl width were estimated using a general linear model, different letters denote significant differences between means with a Benjamini−Hochberg adjusted *P* < 0.05, error bars represent the standard error (*n* = 10). (e) Phenotype of galls developed by *P. brassicae* infected *pOpON::amiRNA‐E2Fa* –DEX and +DEX plants at 26 DAI. (f) Radial cross‐sections of galls formed on the hypocotyls of DEX and mock‐treated *pOpON::amiRNA‐E2Fa* plants at 16 and 26 DAI. The blue colour observed in +DEX sections is the activity of the β‐glucuronidase reporter gene whose expression is driven together with amiRNA‐E2Fa by the same pOpON synthetic promoter. Sections were counter‐stained with Safranin O to allow clear visualisation. Examples of hypertrophied cells are shown with asterisks, scale bars = 200 μm. All experiments were independently replicated at least three times.

In order to gain further knowledge on the involvement of G2‐M checkpoint regulators in *P. brassicae*‐driven cell proliferation within hypocotyls we decided to check levels of key protein factors regulating the duration of the mitotic state. Our transcriptional profiling showed increased levels of the *E2Fa* gene (Figure 1a,b), previously described as a factor stimulating cell proliferation or endoreduplication depending on its interaction with the RBR1 protein (Magyar *et al*., 2012). We examined the E2Fa transcription factor response to infection with a specific antibody (De Veylder *et al*., 2002) and found increased levels of this protein in both the proliferative and the expansive stages of gall formation (Figure 2a). The same membranes were stripped and re‐probed with anti‐RBR1 antibody (Borghi *et al*., 2010). For this protein a similar pattern of upregulation after infection was observed (Figure 2a). Our transcriptional analyses did not show any *RBR1* upregulation (Figure S3), therefore we conclude that its protein abundance is regulated post‐transcriptionally. For E2Fa protein, its elevated levels (Figure 2a) correlated with increased levels of transcript upon infection (Figure 1a,b). We failed to detect any signal after subsequent stripping of our membranes and re‐probing with the anti Phospho‐Rb (Ser807/811) antibody, however independent hybridisation of the E2Fa pull‐down fractions with this antibody showed that elevated pools of RBR1 are phosphorylated (Figure 2a).

To test the importance of the E2Fa increase in blocking cells from quitting the proliferative state during clubroot‐driven gall formation we constructed an artificial miRNA to conditionally silence *E2Fa* gene expression (Schwab *et al*., 2006). Resulting fragments targeting *E2Fa* were cloned into the pOpON2.1 vector (Samalova *et al*., 2005) allowing us to obtain transgenic lines in which silencing of the gene occurred only upon dexamethasone treatment. This way plants could develop normally, without any major disturbance and suppression of *E2Fa* levels occurred only upon treatment with dexamethasone (DEX), which was applied every second day starting from the time seedlings were infected (Figure 2b). Samples were collected at 16 DAI and 26 DAI, plants treated with DEX showed reduced levels of *E2Fa* transcript (Figure 2c) and the above‐ground parts of treated plants were generally smaller (Figure 2b). However, in uninfected plants, there was no significant reduction in hypocotyl width with DEX treatment (Figure 2d). The galls in DEX‐treated plants were significantly reduced in size. At 16 DAI there was some increase in hypocotyl width but significantly less than in the non‐DEX‐treated, infected controls and at 26 DAI the hypocotyl width of infected, DEX‐treated plants was indistinguishable from uninfected plants (Figure 2d,e). Nevertheless *P. brassicae* infection in DEX‐treated plants exhibited the normal symptoms of infection such as the formation of hypertrophied cells and the suppression of xylem development described previously (Malinowski *et al*., 2012) (Figure 2e).

### Maintenance of *P. brassicae*‐driven cell proliferation requires MYB3R4

**Figure 3:**
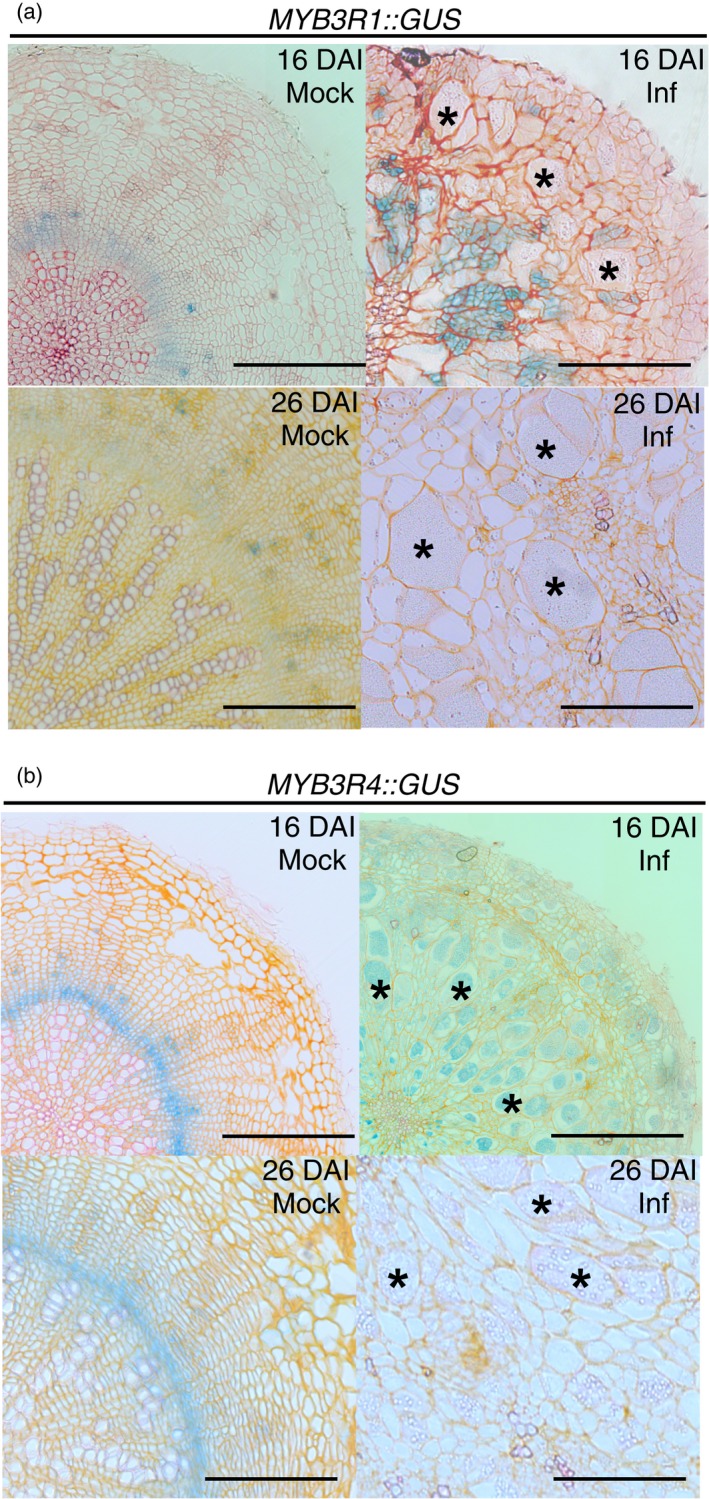
*MYB3R1* and *MYB3R4* are transiently upregulated in response to *P. brassicae* infection. Visualisation of *MYB3R1* and *MYB3R4* promoter activity patterns in MYB3R1::GUS (a) and MYB3R4::GUS (b) reporter lines in radial hypocotyl sections of uninfected (Mock) and infected (Inf) plants at 16 and 26 DAI. Sections were stained for GUS activity, which indicated the extent of the *MYB3R1* and *MYB3R4* induction. Material was counter‐stained with Safranin O. Scale bars represent 200 μm. The experiment was repeated three times and 15 hypocotyls used for each combination, representative examples of each condition are presented. Examples of hypertrophied cells colonised by the pathogen are labelled with asterisks.

It has been shown recently that B‐type cyclin expression is positively regulated by the DREAM complex transcription factor MYB3R4 via the mitosis‐specific activator (MSA) *cis* regulatory motif (Haga *et al*., 2011). The MYB3Rs act in concert with other components of the DREAM complex – E2Fs, DPs, RBR1, ALYs and TCXs. MYB3R4 stimulates proliferation, whereas other DREAM factors repress proliferation and trigger endoreduplication processes. The interaction between DREAM components has not been entirely resolved, some of them appear to have dual functions as both activators and repressors (MYB3R1) and all of these may act simultaneously within the same cell (Kobayashi *et al*., 2015a). We observed increased expression of both *MYB3R1* and *MYB3R4* at 16 DAI (Figure 1a,b), therefore we speculated that these factors could be involved in the observed stimulation of the proliferative state. We decided to further examine spatial expression patterns of the *MYB3R1* and *MYB3R4* genes as well as the possible effects of their knockout. Analysis of non‐infected *MYB3R1::GUS* and *MYB3R4::GUS* reporter lines showed promoter activity that was specific to tissues retaining meristematic character such as the vascular cambium and phloem bundles in mock inoculated hypocotyls at 16 and 26 DAI (Figure 3). This pattern is altered upon *P. brassicae* infection and, at 16 DAI, *MYB3R1* promoter activity is present in regions marking meristematic areas (Fig 3a) typically expanded with *P. brassicae* infection (Malinowski *et al*., 2012) whereas *MYB3R4* promoter activity is present across the entire hypocotyl (Figure 3b). It is worth noting that the GUS staining was present in regions that are not uniformly infected; suggesting a systemic character to the host response to pathogen infection. At 26 DAI the promoter activity of both genes is completely absent in infected hypocotyls.

**Figure 4:**
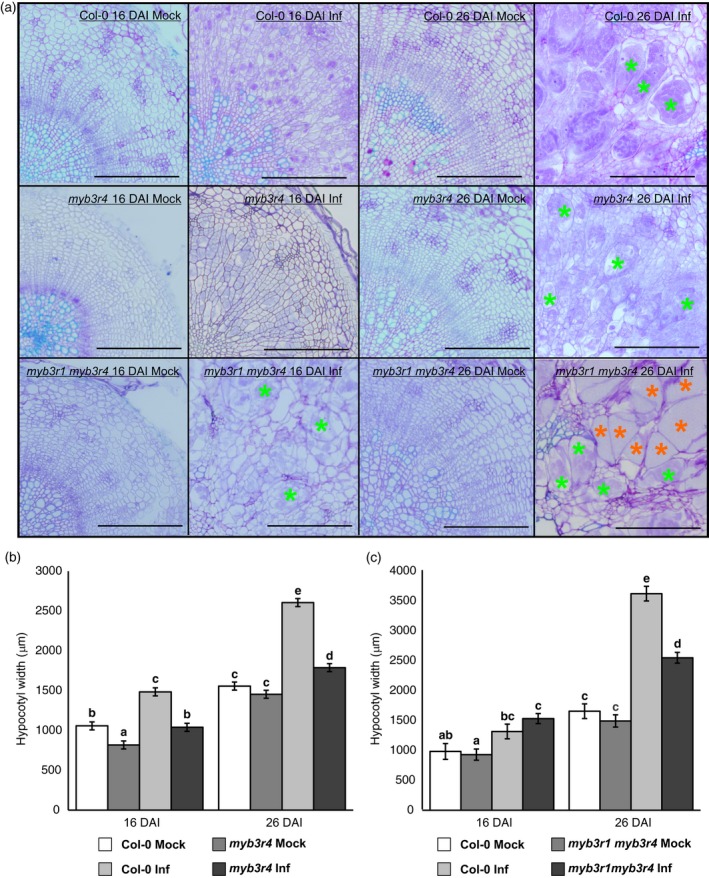
Effects of *MYB3R1* and *MYB3R4* gene knockouts for *P. brassicae*‐driven gall formation. (a) Anatomical changes observed in *myb3r4* single and *myb3r1 myb3r4* double mutants with (Inf) and without (Mock) *P. brassicae* infection 16 and 26 DAI. m*yb3r4* mutant galls were smaller and cells within the gall started expanding earlier than in corresponding Col‐0 controls at the representative time‐points. More pronounced effects on host cell integrity and hypertrophied cell formation were observed in the *myb3r1 myb3r4* double mutant. Green asterisks denote hypertrophied cells filled with secondary plasmodia whereas red asterisks indicate more mature hypertrophied cells containing resting spores. Sections were stained with toluidine blue solution, scale bars = 200 μm. (b, c) Decrease in gall width observed in the *P. brassicae* infected *myb3r4* (b) and *myb3r1 myb3r4* (c) mutants in comparison with Col‐0. Means and variance of hypocotyl width were estimated using a general linear model. Different letters denote significant differences between means with a Benjamini−Hochberg adjusted *P* < 0.05. Error bars represent the standard error (*n* = 10). Experiments were repeated three times.

As *MYB3R4* promoter activity is the pattern most drastically changed with infection we tested the effect of *myb3r4‐1* mutation on gall formation. We found that, similar to the pOpON::amiRNA‐E2FA line, *myb3r4* mutant galls are reduced in size, but that in this mutant, cells within the gall start expanding earlier than in infected Col‐0 controls − at the 16 DAI time‐point (Figure 4). To determine whether MYB3R1 and MYB3R4 TF's act in concert to facilitate proliferation and gall development a double knockout line carrying a rescuing *CDKA;1::KNOLLE* transgene was assessed. The presence of a *KNOLLE* transgene is necessary to rescue cytokinesis defects inherent in the double mutant, as both MYB3R1 and MYB3R4 regulate activation of the *KNOLLE* gene (Haga *et al*., 2007). Hypocotyl swelling in the infected *myb3r1 myb3r4 CDKA;1::KNOLLE* plants was also restricted compared with wild‐type Col‐0 26 DAI (Figure 4b) but the magnitude of gall size restriction was not much greater than that observed in the single *myb3r4* mutant, indicating that MYB3R4 plays a central role in the development of clubroot galls. The accelerated progress of the *P. brassicae* life cycle was more apparent in the *myb3r1 myb3r4 CDKA;1::KNOLLE* plants (Figure 4a).

### *P. brassicae* directly stimulates host cell enlargement

**Figure 5:**
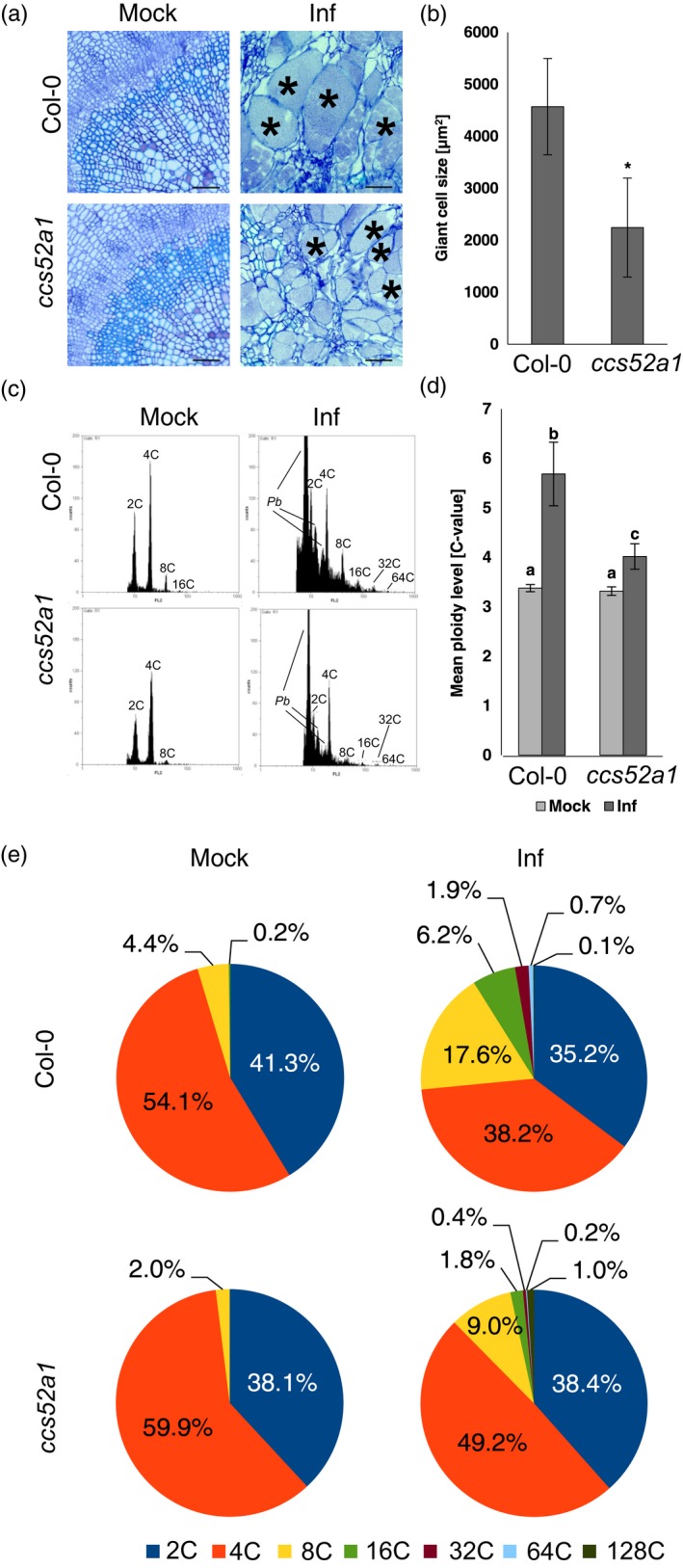
Impaired endoreduplication constrains the size of *P. brassicae*‐induced hypertrophied cells. (a) Toluidine blue stained radial sections of hypocotyls showing representative phenotypes of hypertrophied cells in wild‐type Col‐0 plants and *ccs52a1* mutant plants 26 DAI. Scale bars represent 50 μm, asterisks denote hypertrophied cells. (b) Hypertrophied cell size in the wild‐type Col‐0 plants compared with the *ccs52a1* mutant plants 26 DAI. Values are means of hypertrophied cell area (μm^2^). Calculations were made by overlaying a 3 × 3 grid on each gall section and measuring hypertrophied cells from five randomly chosen squares. Values are means of three independent experiments (10 plants per experiment) ± standard error. Asterisks indicate a statistically significant difference at *P* < 0.05 (Student's *t*‐test). (c) Representative histograms of DNA content determined by flow cytometry in nuclei isolated by from infected and uninfected hypocotyls of Col‐0 and *ccs52a1* plants 26 DAI. (d) Mean ploidy level of nuclei isolated from the hypocotyls of wild‐type Col‐0 plants, compared with *ccs52a1* mutants 26 DAI ± SE. Different letters indicate a statistically significant difference at *P* < 0.05 (Duncan's test), analyses were performed on 12 replicates. (e) Percentage of nuclei with different DNA contents isolated from the hypocotyls of wild‐type Col‐0 plants and *ccs52a1* mutants 26 DAI.

Later stages of gall development (26 DAI) are accompanied by increased host ploidy levels (Figure 1c–e), which coincide with an increased number of hypertrophied cells forming within infected hypocotyls. This finding suggests that the endoreduplication process may be involved in hypertrophied cell formation. Previous reports underline the potential involvement of brassinosterioid‐driven cell expansion in this process (Schuller *et al*., 2014), but so far little attention has been paid to ploidy changes during hypertrophied cell formation. To further understand the contribution of endocytosis in this process we studied the effects of infection on a *ccs52a1* mutant previously characterised as being impaired in endoreduplication (Larson‐Rabin *et al*., 2009). We found that, at 26 DAI, infection still results in increased amounts of 8C to 64C DNA in the mutant nuclei, but they constitute smaller proportions than in the wild type (Figure 5c,d). This difference is reflected in a less pronounced increase in cell area size in pathogen‐colonised cells at 26 DAI (Figure 5a,b). Hypertrophied cells in the *ccs52a1* mutant are, on average, 50.8% smaller than hypertrophied cells in infected Col‐0. Decreased sizes of the hypertrophied cells in *ccs52a1* mutants are borne out in reduced average gall sizes (Figure S4). Impaired potential for the pathogen‐driven enhancement of endoreduplication in *ccs52a1* mutants did not, however, affect the progress of resting spore deposition (Figure 5a). Taken together our data show that the endoreduplication process is an important factor in the formation of hypertrophied cells.

**Figure 6:**
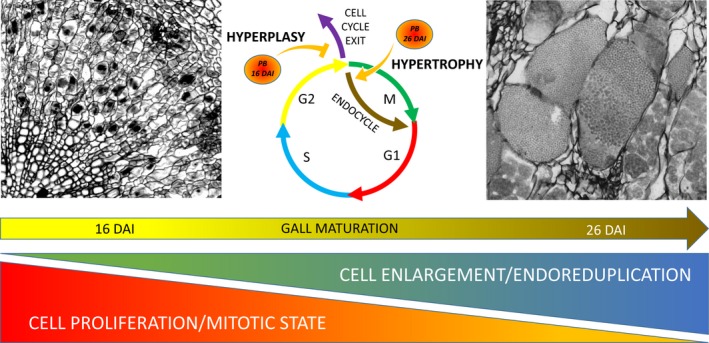
Cell cycle manipulation during clubroot gall development shifts from early inducement of mitotic status towards intensification of the endoreduplication process in later stages. In *P. brassicae*‐infected hypocotyls at 16 DAI we can observe cell proliferation caused by prolonged maintenance of mitotic status through reprograming of the G2−M cell cycle checkpoint and inhibition of cell cycle exit. This situation changes as gall development progresses, cell enlargement at late stages of infection is accompanied by increased endoreduplication. Manipulation of the G2−M checkpoint is again implicated with stimulation of endocycle entry.

Figure 6 summarises, schematically, the changes in host cell cycle behaviour in developing galls over time. We found that host cell cycle is subjected to pathogen‐driven reprogramming that allows for increasing number of cells to be colonised via stimulation of mitotic cell status, whereas later disease stages, associated with resting spore maturation, require cell enlargement that is at least partially facilitated by induction of endoreduplication. The cellular changes induced by *P. brassicae* during both the proliferative and the expansive stages rely on the pathogen's capacity to manipulate the G2−M checkpoint.

## Discussion

### *P. brassicae* infection disrupts mechanisms that regulate the mitotic status of host cells

Our previous work showed that the main cellular consequence of *P. brassicae* infection is a stimulation of existing cambium and phloem meristematic activities in hypocotyls and the upper parts of roots (Malinowski *et al*., 2012). Enhanced levels of cellular proliferation can be achieved via S‐phase stimulation. This scenario has been observed in cell suspension cultures and the involvement of soluble sugars in this process has been described (Riou‐Khamlichi *et al*., 2000; Menges *et al*., 2006). In brief, it has been shown that increased levels of soluble sugars can induce expression of the *CYCD3;1* gene. In response to sugar starvation *CYCD3;1* expression is downregulated in BY2 cells, resulting in G1 arrest. Thus CYCD3;1 controls cell cycle at the G1/S transition and is an important element regulating the switch from cell proliferation to differentiation. *CYCD3;1* gene expression is also induced by cytokinins and, together with the *AINTEGUMENTA* (*ANT*) gene, regulates cell proliferation during secondary root thickening (Randall *et al*., 2015). As increased levels of sucrose accumulation preceded by accumulation of cytokinins at the site of *P. brassicae* infection were reported (Roitsch and Ehneß, 2000), we hypothesised that the observed increase in proliferative cell status was an effect of increased levels of CYCD3;1. This hypothesis was additionally supported by the observation of higher numbers of cells possessing *ANT* promoter activity in *P. brassicae*‐infected hypocotyls (Malinowski *et al*., 2012). However, our transcript and protein level analysis showed that CYCD3;1 is not involved in the observed developmental patterns. Instead, we found substantial alteration of transcripts whose product function is related to the regulation of cell cycle exit. At 16 DAI we can observe upregulation of the G2−M‐specific B‐type cyclin‐dependent kinases (*CDKB*) and B‐type cyclins (*CYCB*); these G2−M‐specific genes are known to be involved in the maintenance of the mitotic phase (Menges *et al*., 2005). Under certain circumstances, the *E2Fa* transcription factor, whose expression was also elevated in hypocotyls of infected plants at 16 DAI, may be involved in the maintenance of proliferation competence (Magyar *et al*., 2012). Our further studies showed that increased levels of E2Fa protein are present both 16 DAI and 26 DAI. In addition, we found that E2Fa is present in a complex with RBR1 protein both at 16 and 26 DAI. It has been shown that, depending on the molecular and cellular context, such a complex can inhibit premature endoreduplication entry (Magyar *et al*., 2012). RBR1 forms a complex with E2Fb that represses cell proliferation. However, in meristematic cells, it forms a more stable complex with E2Fa that inhibits endoreduplication processes. The E2Fa/RBR1 complex suppresses cell differentiation and maintains the proliferative status of cells by repressing expression of *CCS52A* genes (Magyar *et al*., 2012). It has been proposed that the RBR1 protein complexed with E2Fa is resistant to CYCD‐CDK phosphorylation, however in our system the elevated levels of RBR1 were phosphorylated. This does not follow the observation of Magyar *et al*. (2012) but it may be that phosphorylation of RBR1 is affected by other factors associated with *P. brassicae* infection. One possible explanation of the observed difference could be also the existence of a specific factor in the *P. brassicae* genome that can phosphorylate such a complex. In the progress of gall development we can see that mitotic state enhancement takes place only at 16 DAI. By 26 DAI proliferation of host cells stops and mainly local endoreduplication in hypertrophied cells colonised by the pathogen occurs. The *P. brassicae*‐driven transcriptional changes at 16 and 26 DAI show that infection mainly affects the G2−M cell cycle progression checkpoint disturbing, in this way, the important internal regulatory mechanisms necessary for cellular fate specification.

### Inability of plants to respond to clubroot infection with increased E2Fa or MYB3R4 levels reduces cell proliferation within galls

The observed increase in *E2Fa* and *MYB3R4* transcription factor expression coincides with elevated transcript levels of B‐type cyclins and *CDKB1* at 16 DAI. It has been shown that MYB3R4 can bind to MSA motifs in the promoters of some B‐type cyclins (*CYCB*) regulating, this way, the mitotic status of cells (Haga *et al*., 2011; Kobayashi *et al*., 2015b). Conversely, E2Fa interacts with B‐type cyclins at the protein level (Magyar *et al*., 2012). E2Fa and MYB3R4 are likely to be involved in two parallel pathways. Both of these stimulate cell proliferation via B‐type cyclin activation. However, depending on the cellular context, E2Fa may work in combination with different components (Magyar *et al*., 2012). Similarly to *ANT* (Malinowski *et al*., 2012) *MYB3R4* is expressed in all regions of meristematic activity within the hypocotyls (mainly cambium) of non‐infected plants. After infection, this signal spreads across the entire hypocotyl at 16 DAI; this suggests that cells that typically differentiate retain their meristematic activity. We found that galls in *myb3r4* mutants are smaller and, in addition, we saw that infected *myb3r4* cells initiate hypertrophy earlier. This situation indicates that when the proliferative signal is weakened *P. brassicae* infection triggers earlier hypertrophied cell development in the host. This phenomenon was even more pronounced in the *myb3r1 myb3r4* double mutant; suggesting that a failure to adequately stimulate proliferation hastens hypertrophied cell formation.

Earlier onset of cellular hypertrophy, when MYB3R1 or MYB3R4 signalling is compromised, does not compensate for the reduced capacity to increase host cell numbers in terms of gall formation; this underscores the importance of *P. brassicae*'s ability to stimulate host cell proliferation for disease development. These observations are in accordance with the findings from our previous experiments in which cell cycle exit was triggered by chemically inducible *KRP1* gene expression in infected hypocotyls, leading to drastically reduced gall sizes mainly composed of hypertrophied cells (Malinowski *et al*., 2012). Decrease in gall size was also observed when we silenced the *E2Fa* gene in plants.

Based on these results we can say that, for *P. brassicae* infection, differentiation processes in the progeny of cambial cells are delayed through the stimulation of *MYB3R4*, and *E2Fa* TFs and subsequent increase in B‐type cyclins and CDKBs controlling the G2−M checkpoint. As a result of yet‐unknown physiological inputs, proliferation is eventually terminated (by 26 DAI) and individual cells colonised by the pathogen start to expand.

### Host endoreduplication facilitates hypertrophied cell formation

Endoreduplication is an important element of cell fate regulation frequently accompanying cell growth and differentiation in plants. Depending on the physiological context, some cells can replicate DNA without subsequent mitosis. Typically this results in increased DNA content and overall cell size, however certain examples suggest,that final cell size may be regulated at the organ level (Massonnet *et al*., 2011). It has been reported that various pathogens adopt the strategy of inducing host endoreduplication in order to boost host cellular metabolism, facilitating this way their growth and reproduction (Chandran *et al*., 2010; de Almeida‐Engler *et al*., 2012). We found that, during the expansive stage of gall formation, increased proportions of the 8C to 64C polyploid fractions of DNA can be observed. Our RNA‐seq analysis did not reveal a dramatic increase in the expression of factors positively regulating endocytosis; only slight changes in *CCS52a1* and *WEE1* transcript levels were observed. However, by measuring ploidy levels and colonised cell size in the endoreduplication impaired *ccs52a1* mutant (Larson‐Rabin *et al*., 2009), we are able to link restrictions on host polyploidy with reduced cellular hypertrophy and ultimately reduced gall size. Therefore, the process of endoreduplication is bound up with hypertrophied cell expansion. It is possible that *P. brassicae* produces factors directly stimulating endocycling in colonised cells. However, their identification and functional characterisation would require the further development of tools enabling modification of the *P. brassicae* genome.

Based on previous studies of Schuller *et al*. (2014) describing brassinosteroid dependent cell expansion triggered by *P. brassicae*, we can conclude that endoreduplication is not the only process involved in hypertrophied cell formation observed during gall development. It does, however, contribute to the final sizes of colonised cells, influencing in this way the feeding site capacity. Infected mutant plants could still produce galls and form hypertrophied cells, however the size of hypertrophied cells as well as the average gall size of the *ccs52a1* mutant was significantly reduced. Previous studies on root‐knot nematodes (*Meloidogyne* sp.) revealed that failure to induce host endoreduplication during feeding site formation had a critical impact on pathogen development (de Almeida‐Engler *et al*., 2012). Contrastingly, with clubroot disease, impairment of host endoreduplication did not compromise *P. brassicae* life cycle progression but simply reduced the physical space available for replication. The role of particular galls formed in different host−parasite interactions may be strikingly varied, encompassing protective, structural functions to nutritional support for the pathogen. We can assume that, for *P. brassicae* galls, endoreduplication provides more structural value than physiological sink‐related benefits for the pathogen. Despite the fact that in both root‐knot nematode (de Almeida‐Engler *et al*., 1999) and *P. brassicae* gall formation processes we can observe a transient increase in cell proliferation followed by endoreduplication, the patterns of cell cycle reprogramming, and therefore likely the strategies by which they are subverted, differ. For root‐knot nematodes, infection triggers the development of galls composed of multinucleate giant cells resulting from acytokinetic nuclear divisions. It has been shown that early steps of root‐knot gall development are accompanied by upregulation of the S‐phase‐specific *CYCD3;2* kinase and downregulation of *KRP2*, a negative regulator of G1−S progression (Jammes *et al*., 2005). Recent attempts to decrease cell proliferation in developing root‐knot nematode galls by overexpression of the *KRP1* cell cycle inhibitor gene resulted in a reduction in the feeding site size and limitation of nematode development (Vieira *et al*., 2013). For *P. brassicae* we have also observed that the proliferative phase is important for building up the gall to an appropriate size, however manipulation of the proliferative state via an artificial increase in the negative cell cycle regulator *KRP1* did not influence *P. brassicae* life cycle completion (Malinowski *et al*., 2012). Here we also show that impaired endoreduplication in *P. brassicae* infected hosts also do not directly affect the pathogen life cycle progression.

### Future perspectives

The present study demonstrates that the main body of a clubroot gall develops as a consequence of stimulation of the mitotic state of cells at the G2−M checkpoint. In later stages the endoreduplication process is involved in the development of hypertrophied cells. We have established the patterns of cell cycle reprogramming that are triggered by *P. brassicae* in order to increase the size of the feeding site and secure space for subsequent production of resting spores. As *P. brassicae* is a soil‐borne pathogen and obligate biotroph, and its culturing or genetic manipulation presents considerable difficulties, we have not so far been able to identify the pathogen‐related factors involved in modulating the host cell cycle machinery. In the face of these obstacles we hope that future progress may be achieved with the help of *in silico* tools and further exploration and mining of the recently sequenced *P. brassicae* genomes (Schwelm *et al*., 2015; Rolfe *et al*., 2016; Daval *et al*., 2018). For example, this approach may help in the identification of virulence factors that may directly interact with host cell cycle machinery or in the further investigation of the changes to host hormone signalling wrought by the pathogen.

## Experimental procedures

### Biological material

In all experiments *Arabidopsis thaliana* accession Col‐0 was used as a control and the genetic background for all transgenic lines. The *myb3r4‐1* mutant and the reporter *MYB3R4::GUS* lines were characterised in Haga *et al*. (2007), while the *myb3r1‐1 myb3r4‐1 CDKA;1::KN* rescued double mutant line was described in Haga *et al*. (2011). The *ccs52a1* mutant line was previously described by Larson‐Rabin *et al*. (2009). For *E2Fa* gene silencing, a combination of an artificial miRNA technique (Schwab *et al*., 2006) and DEX inducible system (Samalova *et al*., 2005) was used. In brief, WMD3‐designed fragments (wmd3.weigelworld.org) specifically targeting *E2Fa* transcripts were amplified using primers described in Table S2, cloned into the pENTR/D‐TOPO shuttle vector (Invitrogen, http://www.thermofisher.com) and recombined into the pOpON2.1 vector using LR Clonase II (Invitrogen). Plants were transformed by floral‐dip method (Clough and Bent, 1998) and selected on Murashige and Skoog (MS) agar plates supplemented with 75 mg L^−1^ kanamycin according to the protocol described by Harrison *et al*. (2006).

### Growth conditions, infection assay and collection of biological material

All experiments were carried out at a light irradiance of 100 μmol m^−2^ sec^−1^, with a 9 h photoperiod and temperatures of 22/20°C (day/night). Seeds were surface sterilised in 5% commercial bleach, washed in sterile water and germinated *in vitro* on half‐strength MS (Murashige and Skoog, 1962) solid medium containing 0.7% (w/v) agar (BioShop, http://www.bioshopcanada.com) and 1% (w/v) sucrose. Seven days after germination, selected seedlings (with leaf rosette size ranging from 1.2 to 1.5 cm) were transferred to soil substrate (Kronen‐Klasmann Potgrond LT 011). *P. brassicae* inoculum was prepared as described in Malinowski *et al*. (2012) and plants were infected 14 days after germination using 2 ml of spore suspension (1 × 10^−6^ spores ml^−1^). For suppression of the *E2Fa* transcript in the *pOpON::amiRNA‐E2Fa* lines, 2 ml of 10 μm dexamethasone (DEX) (Sigma‐Aldrich, http://www.sigmaaldrich.com) solution was applied every second day starting from the moment when seedlings were infected (14‐day‐old seedlings). For protein and RNA extractions, hypocotyl tissue was collected at 16 and 26 days after inoculation (DAI), these experiments were performed with three independent biological replicates, each with 30 plants per line and per treatment.

### Transcriptome data analysis

RNA‐seq was performed at the Applied Genomics and Analytical Technologies Dept., National Research Council of Canada using the Illumina Hi‐Seq 2500 system. This data has been previously published by Malinowski *et al*. (2016) and raw data from this experiment can be obtained from the European Nucleotide Archive (https://www.ebi.ac.uk/ena) under the identifier PRJEB12261. Significantly differentially expressed genes data were identified using the DESeq2 package in the R statistical environment with False Discovery Rates (FDR) calculated using the qvalue package (Love *et al*., 2014). Heatmaps were generated using the ggplot2 package in R.

### qRT‐PCR analysis

Total RNA was extracted using TRIZOL (Chomczynski and Sacchi, 1987). For the qRT‐PCR template, 2 μg RNA was treated with TURBO DNA‐free Kit (Ambion by Life Technologies, http://www.thermofisher.com) and first‐strand cDNA was synthesised using M‐MLV reverse transcriptase (Promega Reactions were performed using either the Rotor‐Gene 6000 instrument (Corbett Life Science, http://www.qiagen.com) or the LightCycler 480 instrument (Roche, http://www.roche.com) using the SensiMix SYBR No‐ROX kit (Bioline, http://www.bioline.com). Each amplification was carried out with gene‐specific oligonucleotide primers designed using the QuantPrime tool for qPCR (http://quantprime.mpimp-golm.mpg.de/), these primer sequences are detailed in Table S2. Three technical replicates were combined to give an average value for each biological replicate and three independent biological replicates analysed for each condition. Expression levels were calculated relative to the expression of the normalisation gene *18S rRNA* using the comparative quantification method described by Warton *et al*. (2004).

### Protein extraction and immunoblotting

Protein extraction and immunoprecipitation was performed according to Magyar *et al*. (2005) with certain modifications. Hypocotyls were ground in homogenisation buffer containing 25 mm Tris−HCl, pH 7.6; 15 mm MgCl_2_; 150 mm NaCl; 15 mm *p*‐nitrophenylphosphate; 60 mm β‐glycerophosphate; 0.1% Nonidet P‐40; 0.1 mm Na_3_VO_4_; 1 mm NaF; 1 mm phenylmethylsulfonyl fluoride; 1 μm E‐64; cOmplete Mini EDTA‐free protease inhibitor cocktail tablet, 1 per 10 ml (Roche); 0.1% benzonase; 5% ethylene glycol. Equal amounts of total protein (2 mg) were incubated overnight at 4°C with a 1:100 dilution of anti‐E2Fa antibody (Magyar *et al*., 2012) in the same buffer without detergent. Next, 25 μl of protein A agarose beads (Thermo Sci., http://www.thermofisher.com) were added and incubated for 2 h at 4°C on a rotating wheel. Elution was performed by boiling the beads in 5× SDS sample buffer for 10 min at 95°C. The immunoprecipitated proteins were resolved by SDS‐PAGE and protein gel blots were performed. Proteins were detected with the anti‐E2Fa (1:2000 dilution), anti‐RBR1 (1:1000 dilution) (Borghi *et al*., 2010) and the Phospho‐Rb (Ser807/811) monoclonal antibody (Cell Signaling) as primary antibodies. Mouse anti‐rabbit IgG (γ‐chain‐specific) peroxidase (Sigma) was used as a secondary antibody (1:10 000 diluted). To detect the E2Fa complex with RBR1, first the immunodetection was performed with anti‐E2Fa antibody, then the membrane was stripped and re‐probed with anti‐RBR1 antibody. The Phospho‐Rb (Ser807/811) western blots were done on a separately loaded and transferred E2Fa immunoprecipitated fractions. The CYCD3;1 protein detection was performed using 1:1000 dilution of the antibody as described in Kuwabara *et al*. (2011). Experiments were repeated three times.

### Ploidy analysis

Ploidy changes were estimated using a previously described flow cytometry method (Sliwinska *et al*., 2015). For this, hypocotyls from appropriate experimental samples were excised and nuclei were isolated using Galbraith's buffer (Galbraith *et al*., 1983), supplemented with propidium iodide (PI; 50 μg ml^−1^) and RNase A (50 μg ml^−1^). Nuclear DNA content was established in 5000–10 000 nuclei per sample using a CyFlow SL Green (Partec GmbH, Münster, Germany) flow cytometer. Analyses were performed on 12 replicates, using a logarithmic amplification. Histograms were evaluated using the FloMax program (Partec GmbH, Münster, Germany), and the percentage of nuclei with particular DNA contents and the mean C‐value (Lemontey *et al*., 2000) were calculated. Only nuclei having a DNA content of at least 8C were considered to be endopolyploid, as it is not possible to distinguish by FCM analysis the 4C nuclei in cells that have just entered endoreduplication (i.e. being in the G_1_ phase of the first endocycle) from those within cells in the G_2_ phase of the mitotic cycle.

### Anatomy and histology

Histochemical β‐glucuronidase activity assays and microscopic observations were performed as described in Malinowski *et al*. (2012). For anatomical observation and measurements hypocotyls were fixed in Carnoy's solution (absolute ethanol:glacial acetic acid, 3:1) and Technovit embedded and cut as described previously. The obtained 5‐μm sections were stained with 0.05% (w/v) toluidine blue (Sigma‐Aldrich) solution. All sections were mounted in 50% w/v glycerol solution and photographed using the Carl Zeiss AXIO Imager.M2 microscope system. For each sample 6−15 hypocotyls from three independent experiments were sectioned and measured. Hypertrophied cell size area was measured using Image J software (Schneider *et al*., 2012) on toluidine blue stained sections for cells fitting into five randomly chosen squares from a grid dividing the whole section into nine squares. Calculations were made for 15 plants from three independent experiments (five plants per experiment) and analysed by Student's *t*‐test.

## Conflict of interest

We declare no conflict of interest.

## Supporting information


**Figure S1.** CYCD3;1 levels are not elevated in response to *P. brassicae* infection

**Figure S2.** Comparison of gene expression responses to *P. brassicae* infection determined by qPCR and RNA‐seq

**Figure S3.** *RBR1* gene expression is not affected by *P. brassicae* infection

**Figure S4.** Impaired host endoreduplication results in decreased gall size in *P. brassicae*‐infected hypocotyls

**Table S1.** The mean log_2_ ratios between infected and mock‐treated samples list for gene expression sequencing studies presented in Figure 1

**Table S2.** Details of primer sequences used in this study.
