# Supplementary figures and images for: Transcriptional profiling identifies critical steps of cell cycle reprogramming necessary for Plasmodiophora brassicae‐driven gall formation in Arabidopsis
Source: Plant J. 2019 Jan 5;97(4):715–29. doi: 10.1111/tpj.14156 (PMC6850046; doi:10.1111/tpj.14156)

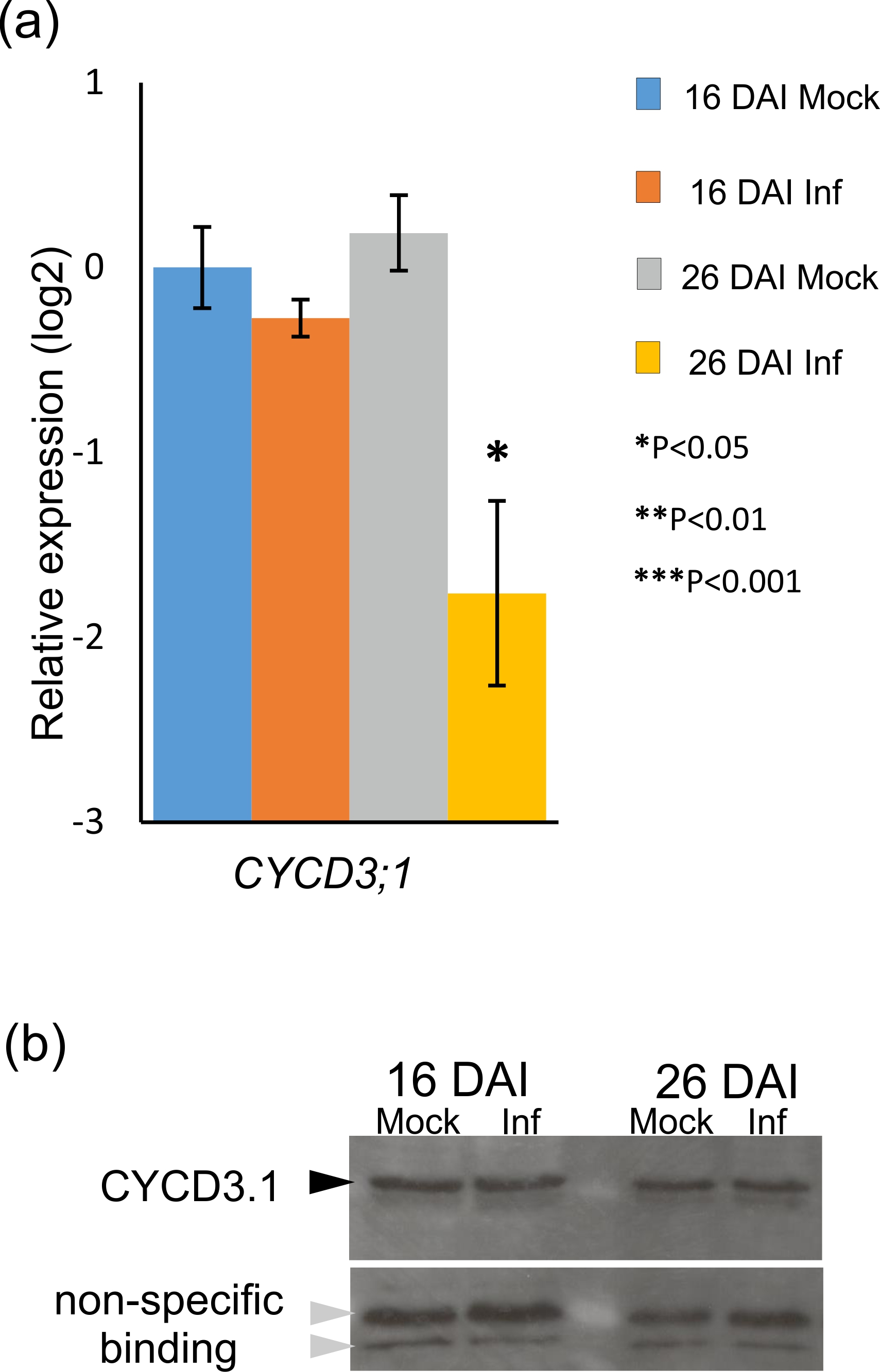

Supplement: Supplementary file 1 — Figure S1. CYCD3;1 levels are not elevated in response to P. brassicae infection [file TPJ-97-715-s001.jpg]

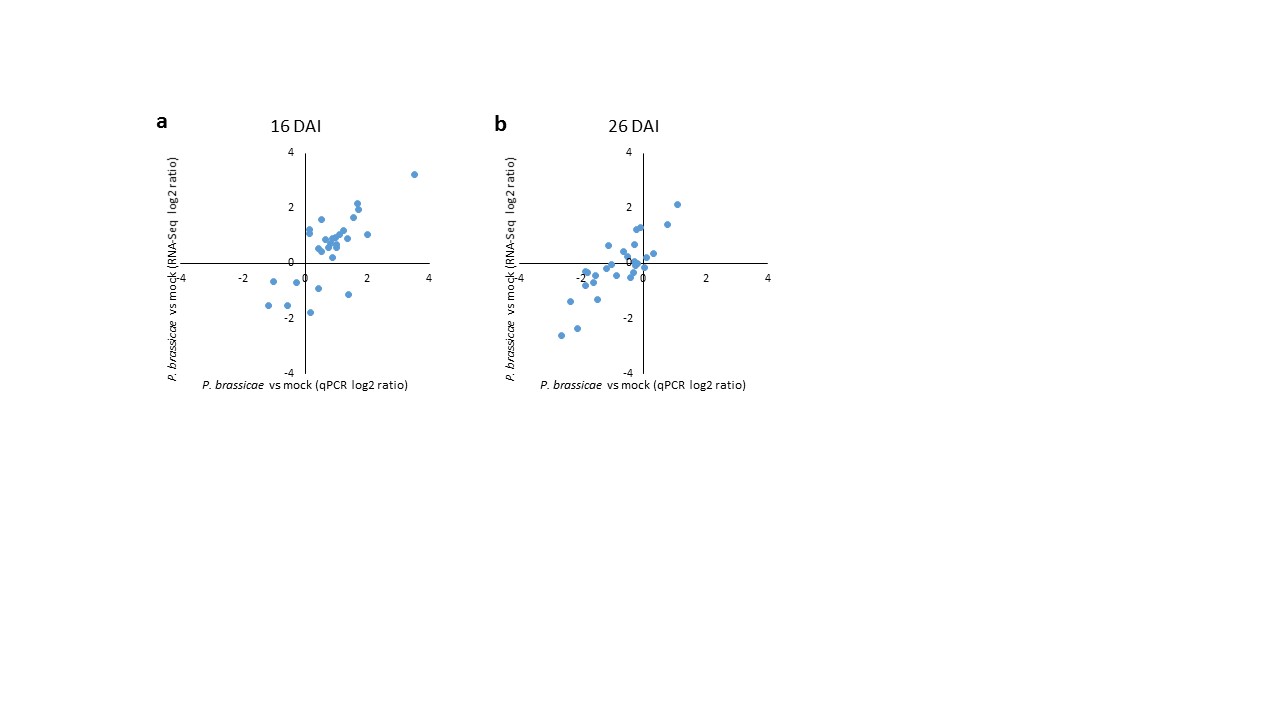

Supplement: Supplementary file 2 — Figure S2. Comparison of gene expression responses to P. brassicae infection determined by qPCR and RNA‐seq [file TPJ-97-715-s002.jpg]

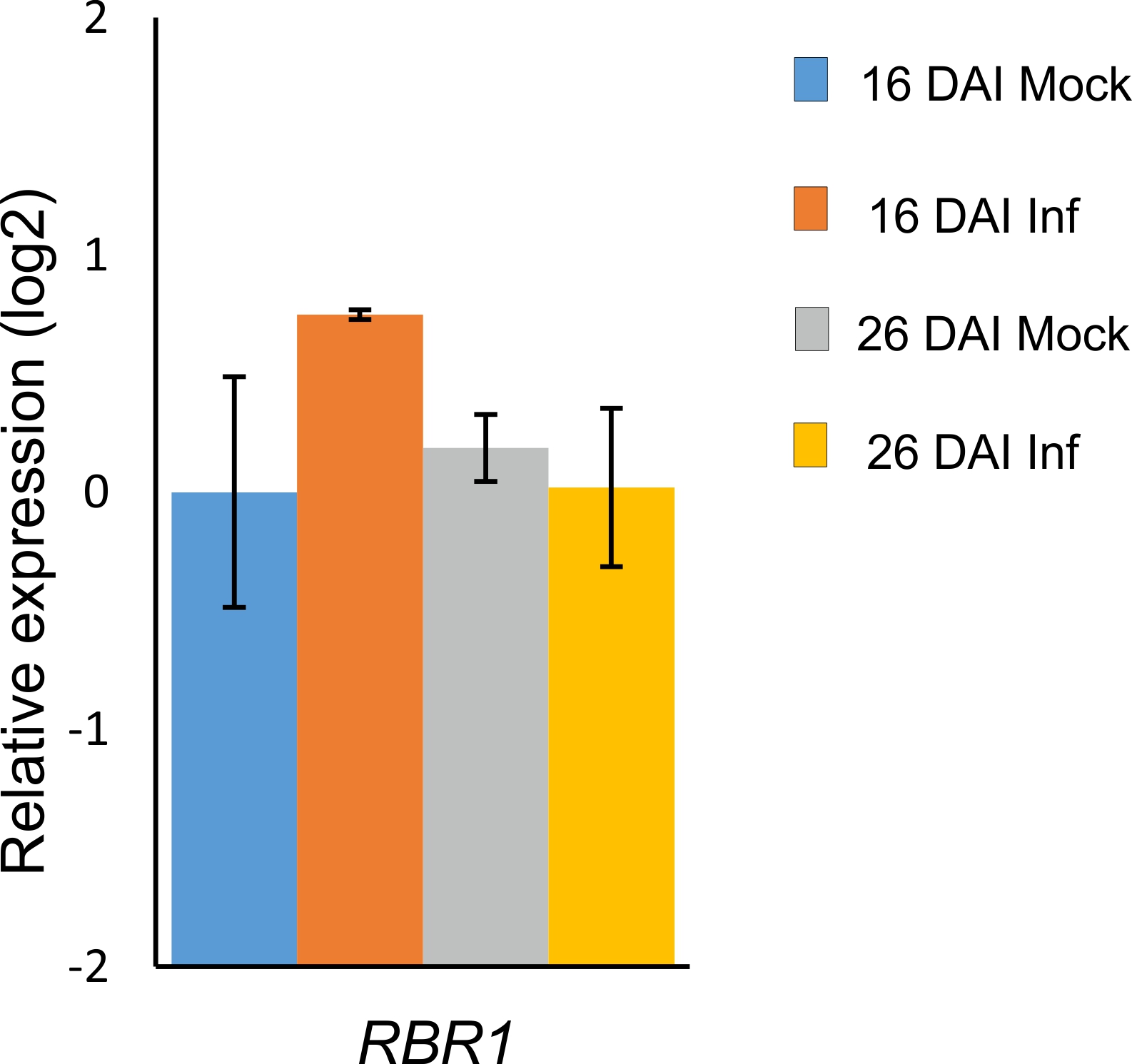

Supplement: Supplementary file 3 — Figure S3. RBR1 gene expression is not affected by P. brassicae infection [file TPJ-97-715-s003.jpg]

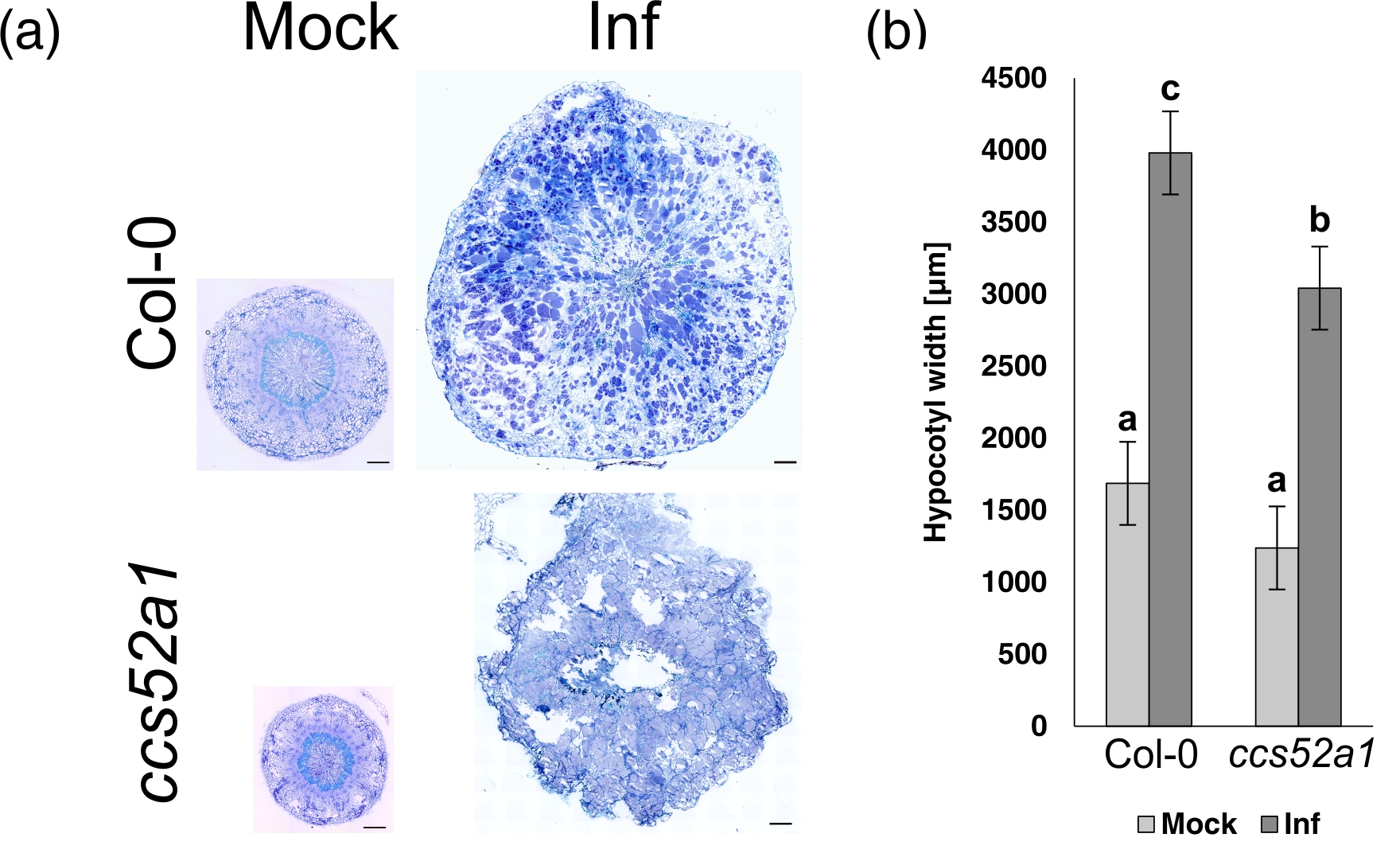

Supplement: Supplementary file 4 — Figure S4. Impaired host endoreduplication results in decreased gall size in P. brassicae‐infected hypocotyls [file TPJ-97-715-s004.jpg]
