# Supplementary material for: Transcriptional profiling identifies critical steps of cell cycle reprogramming necessary for Plasmodiophora brassicae‐driven gall formation in Arabidopsis
Source: Plant J. 2019 Jan 5;97(4):715–29. doi: 10.1111/tpj.14156 (PMC6850046; doi:10.1111/tpj.14156)
Supplement: Supplementary file 6 — Table S2. Details of primer sequences used in this study. [file TPJ-97-715-s006.docx]

**Table S2**

| **RT-qPCR primers** | |
| --- | --- |
|  |  |
| AtCCS52A1 _Fv | CATCTGGTGGAAACGACAATAGGC |
| AtCCS52A1 _Rev | AGCCCATGAACATGAGGAGACC |
| AtCDKB2;1_Fw | TCGACGAGGCTGGAGTTGATCTTC |
| AtCDKB2;1_Rev | TTCGTTTCGCTGGCTCGTACTG |
| AtCDKB2;2_ Fw | TCTTCGTCGGAGCAAAGCAGAC |
| AtCDKB2;2_Rev | AGCTGACCGTGACCACTGAAAC |
| AtCDKD1;1_ Fw | GCCATGATGGACAAGCACCAATG |
| AtCDKD1;1_Rev | ACTGGTGATGGTTGGTCGGTTTG |
| AtCKS2 _Fw | TCGTCCTGAGCCACATATCATGC |
| AtCKS2_ Rev | TAGCAATCTGAGCTTGGTGCTC |
| AtCYCA1;2_ Fw | TCACTGCTGTCTGAGTGTCTGG |
| AtCYCA1;2_ Rev | TGGAGCGTATCGAAGCATAGCG |
| AtCYCA2;1__Fw | AGCCAACTATTTCGCGGAACTAAC |
| AtCYCA2;1_ Rev | AGCGGCAATTAGGGATGGAAGG |
| AtCYCA2;2_ Fw | TGCCACCCGTGAGAAATACAACC |
| AtCYCA2;2_ Rev | CGTTTGGGAGATGTCAGCTTTGC |
| AtCYCA2;4_ Fw | AGATCGCCTCCAAGACCCTTTG |
| AtCYCA2;4 _Rev | ACGAATTTCGGGCTACTTGCTC |
| AtCYCB1;3 _Fw | GCAAGCTACGTGGTGTGTTGAAG |
| AtCYCB1;3 _Rev | ATCAGAGCAACAGCACCTCTTCC |
| AtCYCB1;4 _Fw | TGCGGTTCCATGTGATGCTGAG |
| AtCYCB1;4 _Rev | AAGTCCCAGCTCAGCCAAGTAG |
| AtCYCB2;1 _Fw | GCAGCTCAAGCAGACAAGAAGTG |
| AtCYCB2;2 _Rev | AGTCCACAAGAGCAAGCTCAATC |
| AtCYCB2;3 _Fw | GGCATGTGCGAGAAAGATGGTC |
| AtCYCB2;3 _Rev | ACTTTCTGTGAACTCCTGTGAGC |
| AtCYCB2;4 _Fw | TTCCACTCAGGCTACACCGAAG |
| AtCYCB2;4 _Rev | TCCCTGCCTTGTGATGCAAACC |
| AtCYCD3;1 _Fw | GTCCAAGCTGCGTGATTGATGC |
| AtCYCD3;1_Rev | ACGAACTCGCTGACCACGAATC |
| AtCYCD3;3_Fw | TGTCTGCTTCTGCTTCAGTGTCG |
| AtCYCD3;3 _Rev | TGCTGCTCTTGCACTCTTCTCC |
| AtCYCD4;2 _Fw | TTCCTCTGGAGAGCGAAGAGATCG |
| AtCYCD4;2 _Rev | TCATCTCTCGGTGAGTGTTGTCTC |
| AtCYCD5;1 _Fw | ATGACACCTCCACCGGAAATAGC |
| AtCYCD5;1 _Rev | TTTAGCGCCACTTCCTGAAGCC |
| AtCYCD6;1 _Fw | CGACCCATCTCTGACGTATCTTGC |
| AtCYCD6;1 _Rev | AGGGCTTTGACTGCGGCATATC |
| AtDEL3/E2Ff_ Fw | CCCTTGCTTCTACCTACCAACCAC |
| AtDEL3/E2Ff _Rev | TTCCATGCCTCCGTGAAATGCC |
| AtE2Fa _Fw | ACCTCGTCAGCGAATTTGAAGGG |
| AtE2Fa _Rev | AATAGGCAAGCATGCTGGTGGTG |
| AtE2Fc _Fw | TGCAATCTGAGGAAAGCAGGTTGG |
| AtE2Fc _Rev | ATCTGAGAGCTTCTTGTCGTTCCC |
| AtKRP1 _Fw | ACGGAGCCGGAGAATTGTTTATG |
| AtKRP1 _Rev | CGAAACTCCATTATCACCGACGAC |
| AtKRP3 _Fw | ACAGCTCCAAGTCCAGGTGTTC |
| AtKRP3 _Rev | ATCAGCGGCGGAGGAATTAAGC |
| AtMYB3R1 _Fw | AGCGTGAGATGAAAGCACCTAC |
| AtMYB3R1_Rev | AGCTTTACACAAGACTTCGTCCTC |
| AtMYB3R4 _Fw | AGCACAATACCGGTAAGCCAGTC |
| AtMYB3R4 _Rev | GGGTTGAAACTTGCGCCTTCTC |
| AtSMR1 _Fw | CAAAGAAGGACGAAGGTGATGACG |
| AtSMR1 _Rev | TGTTCTTGGGATGTGGGTGTGC |
| AtSMR6 _Fw | ACCGACAGCGAAGGAAACGAAG |
| AtSMR6 _Rev | TTTCTCGGTGCTGGTGGACATTC |
| AtSMR8 _Fw | GCGGTTTCCGTCAGAATTCCAAG |
| AtSMR8 _Rev | GCACTTCAACGACGGTTTACGC |
| At18sRNA _Fw | CTTTCGATGGTAGGATAGTGGC |
| At18sRNA _Rev | GTCAGGATTGGGTAATTTGCG |
|  |  |
| **amiRNA cloning primers** | |
|  |  |
| I miR-s E2Fa | gaTTCGACGATAATCTAACGCTCtctctcttttgtattcc |
| II miR-a E2Fa | gaGAGCGTTAGATTATCGTCGAAtcaaagagaatcaatga |
| III miR*s E2Fa | gaGAACGTTAGATTAACGTCGATtcacaggtcgtgatatg |
| IV miR* E2Fa | gaATCGACGTTAATCTAACGTTCtctacatatatattcct |
| amiRNA primer A | **cacc**CTGCAAGGCGATTAAGTTGGGTAAC |
| amiRNA primer B | GCGGATAACAATTTCACACAGGAAACAG |
|  |  |
