# Supplementary material for: Transcriptional profiling identifies critical steps of cell cycle reprogramming necessary for Plasmodiophora brassicae‐driven gall formation in Arabidopsis
Source: Plant J. 2019 Jan 5;97(4):715–29. doi: 10.1111/tpj.14156 (PMC6850046; doi:10.1111/tpj.14156)
Supplement: Supplementary file 7 [file TPJ-97-715-s007.docx]

**Figure S1**

CYCD3;1 levels are not elevated in response to *P. brassicae* infection

(a) qRT-PCR assessment of *CYCD3;1* expression in response to *P. brassicae* infection. The experiment was performed with three independent biological replicates, each with 30 plants per treatment, error bars show the standard error. Statistically significant differences (Student’s t-test) between uninfected and infected plants are indicated as follows: * P < 0.05, ** P < 0.01, *** P< 0.001. (b) CYCD3;1 protein levels detected by western blot.

**Figure S2**

Comparison of gene expression responses to *P. brassicae* infection determined by qPCR and RNA-Seq

Log2 ratios between *P. brassicae* infected and mock treated hypocotyl tissue 16 DAI (A) and 26 DAI (B) are plotted for 28 cell cycle genes with qPCR values along the x-axis and RNA-Seq values along the y-axis. These ratios represent the average of three independent replicates for each condition. The genes plotted include all the qPCR data presented in Figure 1. The Pearson correlations between qPCR and RNA-Seq results are 0.74 for 16 DAI and 0.82 for 26 DAI.

**Figure S3**

*RBR1* gene expression is not affected by *P. brassicae* infection

qRT-PCR measurement of *RBR1* gene expression upon *P. brassicae* infection. The experiment was performed with three independent biological replicates, each with 30 plants per treatment, error bars show the standard error. No statistically significant differences between uninfected and infected plants were detected.

**Figure S4**

Impaired host endoreduplication results in decreased gall size in *P. brassicae* infected hypocotyls

(a) Representative radial sections of toluidine blue stained Col-0 and *ccs52a1* hypocotyls 26 DAI with (Inf) and without (Mock) *P. brassicae* infection. Scale bars represent 200 µm. (b) Gall size of Col-0 and *ccs52a1* hypocotyls 26 DAI. Means and variance of hypocotyl width were estimated using a mixed linear model. Different letters denote significant differences between means with a Benjamini-Hochberg adjusted p-value < 0.05. Error bars represent the standard error (n=15).

**Table S1**

The mean log2 ratios between infected and mock treated samples are listed for each gene from the heatmap in Figure 1. The False Discovery Rate (Storey, 2003) is included in parentheses.

**Storey, J.D.** (2003) The positive false discovery rate: a Bayesian interpretation and the q -value. *Ann. Statist.*, **31**, 2013-2035.

**Table S2**

Details of primer sequences used in this study.
